# Comparing Fenton and INTERGROWTH‐21st Growth Charts in Assessing Preterm Infant Growth Below 33 Weeks' Gestation

**DOI:** 10.1002/pdi3.70051

**Published:** 2026-04-28

**Authors:** Vitor dos Santos Andrade, Juliana Dantas de Araújo Santos Camargo, Sávio Ferreira Camargo, Amanda Karoline da Costa Bezerra, Raionara Cristina de Araújo Santos, Cijara Leonice de Freitas, Camiliane Azevedo Ferreira, Ricardo Ney Cobucci, Claudia Rodrigues Souza Maia

**Affiliations:** ^1^ Januário Cicco Maternity School Brazilian Company of Hospital Services Natal Rio Grande do Norte Brazil; ^2^ Postgraduate Program in Health Science Federal University of Rio Grande do Norte Natal Rio Grande do Norte Brazil; ^3^ Department of Public Policy Management Federal University of Rio Grande do Norte (UFRN) Natal Rio Grande do Norte Brazil; ^4^ Department of Pediatrics Federal University of Rio Grande do Norte (UFRN) Natal Rio Grande do Norte Brazil

**Keywords:** anthropometry, growth charts, infant, premature

## 1 Introduction

Prematurity remains a leading cause of neonatal morbidity and mortality worldwide, with an estimated 15 million infants born preterm (< 37 weeks) each year, and with substantial regional variation in prevalence across countries [1]. Risk factors for preterm birth are multifactorial and include both biological and social determinants, underscoring the need for accurate monitoring and follow‐up of this vulnerable population [2].

Birth weight and length are strong predictors of short‐ and long‐term outcomes in preterm infants, and accurate postnatal growth monitoring is essential for their care. Two anthropometric tools commonly used in clinical practice are the Fenton preterm growth charts and the INTERGROWTH‐21st newborn standards, developed as an international prescriptive standard derived from healthy, low‐risk populations [3, 4].

Previous studies in Brazil have compared these charts at birth, but evidence remains limited regarding their concordance during the postnatal period, particularly at hospital discharge and the first outpatient follow‐up, in high‐risk tertiary care settings [5]. This study compares the anthropometric classification of preterm infants using the Fenton and INTERGROWTH‐21st charts at three clinically relevant time points: birth, hospital discharge, and first outpatient follow‐up.

## 2 Methods

This prospective cohort study was conducted at a referral maternity hospital specializing in high‐risk pregnancies and a university‐affiliated outpatient clinic in Northeast Brazil. In 2019, the institution recorded a total of 3561 births, among which 292 infants presented with gestational ages ranging from 27 to 33 weeks. This cohort is part of the national Avaliação BRAsileira do Crescimento de Ex‐prematuros (ABRACE) network, which aims to investigate post‐discharge growth patterns in very low birth weight preterm infants across Brazil. The ABRACE initiative faced interruptions due to the COVID‐19 pandemic, resulting in the absence of a unified protocol publication.

The convenience sample comprised preterm neonates born between January 2019 and February 2020, characterized by gestational ages of 27–33 weeks and/or birth weights below 1500 g (including two infants with a birth weight of 1500 g but gestational age of 33 weeks). Exclusion criteria included transfer to other healthcare facilities, mortality during hospitalization, significant congenital malformations or chromosomal syndromes, and late admissions from alternative maternity units. Twins were included in the study. The final sample consisted of 82 neonates at birth, 80 at discharge, and 70 at the first outpatient visit, yielding an 85% follow‐up rate, with losses primarily due to missed appointments and challenges in post‐discharge contact.

Anthropometric measurements were obtained by trained examiners following standardized procedures consistent with the INTERGROWTH‐21st and ABRACE protocols. Birth weight was measured immediately after delivery using a calibrated digital scale and recorded in grams. At hospital discharge and during the first outpatient visit, weight was again measured on the same scale with the infant undressed. Length was assessed in the supine position using the same infantometer at all time points. Two trained examiners performed each measurement: One positioned the head against the fixed cephalic board while the other extended the legs to align the feet with the movable end. Readings were taken to the nearest millimeter and recorded in centimeters. All measurements were first recorded on the standardized ABRACE form and subsequently entered into the Fenton (2013) and INTERGROWTH‐21st calculators (using sex and gestational age) to generate *z*‐scores for weight and length at birth, discharge, and follow‐up.

Continuous variables were assessed for normality using the Shapiro–Wilk test and summarized as mean ± standard deviation (SD) or median and interquartile range (IQR, 25th–75th percentile); categorical data were reported as counts and proportions. Paired comparisons employed paired *t*‐tests (for normally distributed data) and Wilcoxon signed‐rank tests (for non‐normally distributed data). Corresponding effect sizes were derived using Cohen's *d* (for parametric analyses) or the matched rank biserial correlation (for non‐parametric analyses), ensuring standardized interpretation of the magnitude of observed diferences. Power analyses were conducted utilizing the R software (R version 4.5.1), and overall statistical analyses were performed using JASP version 18.3.0, with significance established at a two‐tailed *p*‐value of less than 0.05. Ethical approval was obtained (CAAE: 97359518710015327), and written consent was acquired from all parents or guardians.

## 3 Results

The study included 82 preterm neonates (49 females, 33 males) with a median gestational age of 31 weeks (IQR, 29–32), mean birth length of 38.9 cm (SD 3.2), and mean birth weight of 1391 g (SD 355). The majority of the infants were classified as appropriate for gestational age (AGA; *n* = 73), whereas a small proportion were classified as small for gestational age (SGA; *n* = 9). Deliveries were predominantly via cesarean section (*n* = 51), with 30 recorded as vaginal births. Maternal preeclampsia was present in 28 cases, and 13 neonates had Apgar scores of less than 7 at five minutes. Common neonatal morbidities included periventricular hemorrhage (*n* = 24), respiratory distress syndrome (*n* = 23), late‐onset sepsis (*n* = 14), and necrotizing enterocolitis (*n* = 6).

Feeding strategies during hospitalization included exclusive breastfeeding (*n* = 37), mixed feeding (*n* = 27), and exclusive formula feeding (*n* = 11). At discharge, the median corrected gestational age was 37 weeks (IQR, 36–39), with a median length of 44 cm (IQR, 42–45) and a median weight of 1998 g (IQR, 1880–2122). At the first outpatient visit (*n* = 69), the median corrected gestational age was 45 weeks (IQR, 44–46), the median length was 50 cm (IQR, 48–53), and the mean weight was 3630 g (SD 771).

At birth, length *z*‐scores were comparable across growth charts, while weight *z*‐scores were consistently lower according to the Fenton chart (−0.3 *vs.* −0.1, *p* = 0.001, rank‐biserial correlation = 0.5). At discharge, both length (−1.9 *vs.* −1.7, *p* = 0.035, rank‐biserial correlation = 0.3) and weight (−2.2 *vs.* −1.6, *p* = 0.001, rank‐biserial correlation = 0.9) *z*‐scores significantly differed between the charts. At the first follow‐up, length *z*‐scores remained lower with the Fenton chart (−2.0 *vs.* −1.8, *p* = 0.001, Cohen's *d* = 0.4), with an even more pronounced difference in weight (−1.8 *vs.* −1.3, *p* = 0.001, Cohen's *d* = 3.1). The comparison of weight *z*‐scores at the initial visit demonstrated a post hoc power of 86% at an alpha level of 0.05 (Table 1).

**TABLE 1:** Mean *z*‐scores for weight and length using the Fenton and INTERGROWTH‐21st charts at birth, discharge, and first outpatient visit.

Time point	Fenton length (*z*)	Intergrowth length (*z*)	*p* value	Fenton weight (*z*)	Intergrowth weight (*z*)	*p* value
Birth	−0.4 (−1.0, 0.4)	−0.3 (−1.1, 0.2)	0.293	−0.3 (−1.1, 0.3)	−0.1 (−0.7, 0.8)	**0.001**
Hospital discharge	−1.9 (−3.0, −1.1)	−1.7 (−3.4, −0.7)	**0.035**	−2.2 (−3.2, −1.7)	−1.6 (−2.6, −1.0)	**0.001**
First visit	−2.0 ± 1.4	−1.8 ± 1.8	**0.001**	−1.8 ± 1.3	−1.3 ± 1.4	**0.001**

*Note:* Differences between groups were assessed using paired *t*‐tests or Wilcoxon signed‐rank tests, as appropriate. Continuous data are presented as mean ± standard deviation or median and interquartile range (25th–75th percentile). Bold *p* values indicate statistical significance (*p* < 0.05).

## 4 Discussion

This study identified notable differences in anthropometric classification of preterm infants using the Fenton and INTERGROWTH‐21st charts. Weight *z*‐scores were consistently lower with the Fenton chart, suggesting higher sensitivity to growth deficits. Length measurements were similar at birth but diverged at discharge and follow‐up, reflecting differences in postnatal growth interpretation.

These discrepancies align with differences in chart construction: The Fenton chart is based on fetal growth data from high‐income countries, whereas INTERGROWTH‐21st derives from an international cohort of healthy, low‐risk pregnancies designed as a prescriptive standard [3, 4]. Our analyses used the 2013 Fenton chart [3]; with the 2025 updated Fenton curves, observed differences may change, highlighting the need for further validation in diverse populations.

Clinically, chart selection affects the identification of extrauterine growth restriction and subsequent nutritional interventions. Although the Fenton chart flagged more infants as growth‐restricted, this does not imply superiority. INTERGROWTH‐21st may underestimate malnutrition in settings with postnatal nutritional deficits, whereas Fenton may overdiagnose restriction in heterogeneous populations [3, 4, 5]. These findings emphasize interpreting growth alongside clinical and nutritional context rather than relying solely on one chart.

Strengths of the study include prospective data collection, standardized protocols, and comparison of two widely used international charts. This study has limitations, including its small single‐center sample, loss to follow‐up, and the lack of data on head circumference and nutritional details, which are essential for understanding growth trajectories.

## 5 Conclusion

Chart selection substantially affects anthropometric classification in preterm infants. The Fenton chart may detect more growth restriction, whereas INTERGROWTH‐21st provides a prescriptive framework with broad global applicability. Both charts should be interpreted cautiously within the clinical context, and further research is needed to evaluate their performance across diverse populations.

## Author Contributions

**Vitor dos Santos Andrade:** Investigation, data curation, writing – review and editing. **Juliana Dantas de Araújo Santos Camargo:** Data curation, conceptualization, methodology, formal analysis, writing – original draft, writing – review and editing, supervision. **Sávio Ferreira Camargo:** data curation, investigation, writing – review and editing. **Amanda Karoline da Costa Bezerra:** investigation, data curation, writing – review and editing. **Raionara Cristina de Araújo Santos:** investigation, writing – review and editing. **Cijara Leonice de Freitas:** investigation, writing – review and editing. **Camiliane Azevedo Ferreira:** investigation, writing – review and editing. **Ricardo Ney Cobucci:** conceptualization, supervision, writing – review and editing. **Claudia Rodrigues Souza Maia:** conceptualization, supervision, funding acquisition, writing – review and editing.

## Funding

This study received funding from the Coordination for the Improvement of Higher Education Personnel (CAPES).

## Conflicts of Interest

The authors declare no conflicts of interest.

## Data Availability

The data that support the findings of this study are available from the corresponding author upon reasonable request.

## References

[1] E. O. Ohuma , A.‐B. Moller , E. Bradley , et al., “National, Regional, and Global Estimates of Preterm Birth in 2020, With Trends From 2010: A Systematic Analysis,” Lancet 402, no. 10409 (2023): 1261–1271, 10.1016/S0140-6736(23)00878-4. 37805217

[2] I. Mitrogiannis , E. Evangelou , A. Efthymiou , et al., “Risk Factors for Preterm Birth: An Umbrella Review of Meta‐Analyses of Observational Studies,” BMC Medicine 21, no. 1 (2023): 494, 10.1186/s12916-023-03171-4. 38093369 PMC10720103

[3] T. R. Fenton and J. H. Kim , “A Systematic Review and Meta‐Analysis to Revise the Fenton Growth Chart for Preterm Infants,” BMC Pediatrics 13, no. 1 (2013): 59, 10.1186/1471-2431-13-59. 23601190 PMC3637477

[4] J. Villar , L. Cheikh Ismail , C. G. Victora , et al., “International Standards for Newborn Weight, Length, and Head Circumference by Gestational Age and Sex: The Newborn Cross‐Sectional Study of the INTERGROWTH‐21st Project,” Lancet 384, no. 9946 (2014): 857–868, 10.1016/S0140-6736(14)60932-6. 25209487

[5] C. M. Barreto , M. A. L. Pereira , A. C. B. Rolim , S. A. Abbas , D. M. Langhi Junior , and A. M. N. D. Santos , “Incidence of Small for Gestational Age Neonates, According to the Fenton and Intergrowth‐21st Curves in a Level II Maternity,” Revista Paulista de Pediatria 39 (2021): e2019245, 10.1590/1984-0462/2021/39/2019245. 32638944 PMC7333938

